# Diseases of Children

**Published:** 1894-08-04

**Authors:** 


### MEDICAL PROGRESS.

DISEASES OF CHILDREN.—*Continued.*

**Diphtheria and Croup.**—At the International Medical Congress a discussion took place on diphtheria and croup.[23] Professor Escherich held that secondary infections in diphtheria due to pyogenic organisms frequently determined the clinical character of a particular case. Dr. Baginsky had found that the renal complications in diphtheria were dependent on a glomerular nephritis with considerable epithelial desquamation. Dr. Heubner had come to the conclusion that the serum treatment had no influence on the disease. Dr. Concetti supported the local treatment by antiseptics, and Dr. Soltman referred to the necessity for continuing disinfection of the mouth for some time after the disappearance of all morbid products. The advisability of this measure is illustrated in a fatal case of diphtheria recorded by Belfanti,[24] in which the source of infection was traced to a brother who had suffered from the disease seven months previously and had recovered. The tonsils were still enlarged and chronically inflamed, and Loeffler's bacilli were found in the tonsillar exudation. That these were still active at that remote period was proved by cultivation experiments. Three months later the bacilli were still present, but in a state of great attenuation. Dr. Emmett Holt[25] summarises the treatment of diphtheria as follows: (1) Germicidal treatment, preferably by the use of strong hydrochloric acid, used early to be effectual; (2) local cleanliness by the application of a weak antiseptic solution to the throat; (3) nasal syringing with the same solution in every case where there is a discharge from the nose; (4) alcoholic stimulants begun as soon as the first systemic effects of the poison are seen, and in very severe cases pushed to the point of tolerance; (5) calomel fumigations as soon as laryngeal symptoms appear; and (6) intubation in laryngeal cases which are not relieved by fumigation. Ehrlich, Kossel, and Wasserman[26] have treated 220 cases of diphtheria with the serum of goats artificially rendered immune by the administration of increasing doses of dead diphtherial cultures. The results obtained in those in whom treatment was commenced on the first or second day of the illness are extremely encouraging. In the others the mortality was not apparently diminished. Bianchini[27] applies fomentations of a 2 per cent. solution of carbolic acid round the neck of patients suffering from diphtheria, so as to act on the micro-organisms in the local lesion by the antiseptic absorbed through the skin and through the mouth. At the same time he applies to the throat a paint containing salicylic acid, resorcin, and alcohol. Of 54 cases thus treated, 52 recovered. Dr. W. A. Greet[28] has found acetous vapour useful. He takes a quart of malt vinegar in a steam kettle, and keeps the vapour from this constantly about the patient. Rapid improvement followed on this treatment in a case of laryngeal and tonsillar diphtheria. Dr. C. L. Scudder,[29] after performing tracheotomy for diphtheria in a boy aged four years, with only temporary relief to the breathing, proceeded to curette the inside of the trachea with a dull wire intrauterine curette. Several pieces of membrane were removed, and relief to the breathing followed, the child eventually making a good recovery. Sublimed sulphur has been found a very useful local application in cases of diphtheria by Dr. R. F. Fraser.[30] He blows the powder on to the throat, and sometimes burns a small amount of sulphur in the room. This remedy was originally recommended by Lagauterie in 1866, but its use has never become general. Since Dr. Fraser's communication appeared, Professor Baümler[31] writes, that after seven years' experience he has got better results from sulphur than from any other local application. The benefit is most marked in those cases in which the disease is limited to the fauces.

**Acute Pneumonia.**—Dr. T. S. Southwood[32] has contributed a valuable paper on this subject. The lobar form in most cases runs a typical and well-recognised course; but there are also irregular varieties, which he groups under the following headings: (1) abortive, (2) wandering, (3) gastric, and (4) cerebral. The abortive type is characterised by the sudden subsidence of all symptoms in twenty-four to thirty-six hours, and is caused by a local congestion which has not gone on to consolidation. Wandering pneumonia is a term applied to cases in which fresh areas of inflammation and consolidation appear, and delay the crisis. The other forms are those in which the gastric or cerebral symptoms predominate, and tend to direct attention away from the pulmonary condition. They are most apt to mislead when the pneumonia is deeply seated, and the physical signs are few or entirely absent. In all cases, however, the continuous high temperature, the pulmonary embarrassment, the cough, and the characteristic respiratory rhythm are sufficiently diagnostic. As a rule the prognosis in lobar pneumonia is very good, the mortality being about 2 per cent. Broncho-pneumonia is a much more fatal disorder, and is apt to attack children weakened by constitutional disease or acute illness. The primary change is in the bronchi, but inflammatory areas soon arise in the adjacent alveoli and intra-alveolar connective tissue. The lungs are further crippled by the development of atalectasis, congestion, and emphysema. With such varied pathological changes no definite course can be laid down, and the physical signs vary in each case from day to day. It is only by careful observation of the patient's condition as regards temperature, cough, dyspnœa, and cardiac action, along with frequent examination of the chest, that an accurate diagnosis can be made. The mortality is variously estimated at from 30 to 70 per cent. In cases showing a continuous high temperature the bath or wet pack is the best treatment, being stimulant as well as antipyretic. The bath further produces a feeling of comfort in the patient, and calms the nervous system, thus removing delirium and favouring sleep.

**Infant Feeding.**—On this subject Dr. Louis Fischer[33] gives some good practical directions. Every mother should nurse her child, except when she is suffering from exhaustion, tuberculosis, or some marked nervous condition. Whenever children remain below normal as regards weight they must be weaned. Nursing should be stopped at once on the recurrence of pregnancy but not of menstruation. The weaning process should be gradually carried out from the eighth to the tenth month. No food of a starchy nature is to be given to infants during the first three months of life. The mixed milk of a dairy is better than the so-called nursery milk, which is supposed to be the product of one animal. Dr. W. P. Northrup[34] describes the practical working of a milk laboratory which has been established in New York. The object is to modify fresh cow's milk so that the relative proportions of fat, sugar, and albuminoids may be as nearly as possible the same as they are in good average breast milk. As regards the milk supply, they select those cows whose milk is well suited to the raising of calves, although possibly not to the production of butter. The milk having been speedily and carefully conveyed to the laboratory, is placed in the separator, which by centrifugal action removes all impurities from the milk and separates it into (1) the cream and fat; (2) a clean, separated milk without fat. For the production of the compound resembling breast milk the following are also employed, namely—(3) distilled water; (4) lime water; (5) a 20 per cent. solution of milk sugar. These are then mixed together in the proportion determined on as suitable for the individual case, and can be varied as required. A similar laboratory has been established in Boston by Dr. Rotch.[35] The artificially changed milk, before being distributed to families, is "pasteurized;" that is to say, heated to a temperature of 167 degrees Fahr. for twenty minutes. If the milk is to be kept for more than twenty-four hours a higher temperature is required, namely, 212 degrees Fahr., but this changes somewhat the taste of the milk and coagulates the albuminoids. Messrs. R. Lezé and E. Hilsont[36] describe a practical test of the quality of milk by means of rennet. Good fresh milk will coagulate at a temperature of 55 degrees C. in from three to four minutes, under the influence of one part of commercial rennet diluted with 1,000 parts of water. Milk which takes more than four or less than two minutes to coagulate under this test is not suitable for domestic purposes. Dr. Bellot[37] has drawn attention to the disturbances which follow from overfeeding in infants and children. Hepatic or renal colic is not very rare. Gastro-intestinal troubles very often lead to skin disorders, such as urticaria, eczema, acne, or ecthyma. In some cases these are due to ptomaines absorbed directly from the intestine, in others to reflex influences, and in others to the irritation caused in the skin by the excretion of volatile fatty acids. It is specially to be noted that digestive derangement is not at all necessary for the production of skin diseases in an over-fleshy or over-fed child.

**Rachitis.**—The spasmodic affections associated with rickets were under discussion at the Medical Congress.[38] Dr. Comby was of opinion that the digestive troubles accompanying rickets were responsible for the convulsions, which were caused by the absorption of toxins from the alimentary canal. Dr. Chaumier had made a special study of rickets in Italy, and had come to the conclusion that it was due to a microbe as yet undiscovered. Cases of apparent heredity were explained by the fact that the germs of the disease might exist in houses for a long time. Dr. Borelli considered that the conditions of intra-uterine life had an important predisposing influence. In a clinical lecture on rickets Professor A. E. Hoadley[39] remarks that he invariably finds a history of too frequent feeding in cases of this affection, and as a result that the process of digestion is interrupted before all the food elements have been sufficiently changed to admit of assimilation. Treatment must be directed to remedy existing evils and prevent their repetition in the future. The alimentary canal is to be washed out thoroughly by means of seidlitz powders, one every hour in half a glassful of water, until all fæcal matter is evacuated and the medicine passes through mixed only with intestinal mucus. From two to four powders will suffice. An intestinal disinfectant, such as salol, is then employed, one grain of which is given every two hours until twelve have been taken. The next step is to establish a uniform and rather long interval between meals. Feeding is to be carried out four times a day, and absolutely nothing but water is to be allowed between meals.

**Intussusception.**—Mr. A. E. Barker[40] has published the notes of four additional cases treated by him, and given a table of 25 cases admitted into the surgical wards of University College Hospital. He formulates his conclusions as follows: (1) in all cases of intussusception in children, injection of water or manipulation should be at once resorted to if the patient is seen within a few hours of the onset of the strangulation. (2) If these means fail after a fair trial, not too much prolonged, laparotomy should be done at once as the safest treatment. (3) There is a certain proportion of cases among all the varieties of intussusception which no amount of injection will relieve, or in which injection would be dangerous, and these can only be dealt with by opening the abdomen. Dr. Creswick Williams[41] has recorded a case of intussusception in a child of eight months which he treated successfully by generating carbon dioxide within the bowel. He injected *per rectum* a drachm and a half of citric acid dissolved in water, followed by a solution of two drachms of bicarbonate of soda. Carbonic acid gas was quickly generated, the abdomen became distended, and the intussusception was permanently reduced.

**Acute Meningitis.**—In one case, probably tubercular, Dr. Wallis Ord and Mr. Waterhouse[42] have operated successfully. The patient was a girl of five years, suffering from signs of meningitis with commencing optic neuritis. As she seemed to be passing rapidly into a condition of coma from intra-cranial pressure, the skull was trephined midway between the external occipital crest and the mastoid process, and a drainage tube was introduced into the subarachnoid space between the cerebellum and the medulla. The patient made a good recovery. In a case of chronic hydrocephalus, in an infant nine weeks old, Mr. B. Baskett[43] opened and drained the left ventricle with marked improvement for some weeks, after which suppuration set in and the child died.

**Therapeutic Notes.**—Patterson[44] recommends in the summer diarrhœa of children sulphate of magnesium in teaspoonful doses daily until the discharges become yellow. Sevestre[45] has found citrate of caffeine very valuable in the cardiac adynamia accompanying acute febrile disorders, or collapse from any cause. It is best administered hypodermically in three-grain doses twice a day. Bromoform has been employed with good results in whooping-cough by Pelicer.[46] The dose is one drop for each year of the patient's life, given four times a day, and increased steadily as required. In the same disease Raubitschek[47] has found intrapharyngeal applications of perchloride of mercury solution (1-1,000) very useful. In the treatment of chronic rheumatism, Jules Simon[48] finds colchicum superior to any other drug. He gives ten drops of the tincture one hour before dinner for eight days, and then allows an interval of a week before resuming the treatment. Mann[49] has successfully treated a case of hæmophilia with the red marrow of bone. The form of administration was a glycerine extract of the heads of long bones from recently killed calves. Iron, arsenic, and cod-liver oil had been previously employed without benefit in this case, a boy aged twelve years, but after taking the bone marrow his blood corpuscles increased from 3,800,000 to 4,400,000, and his colour improved greatly.

[23] B.M.J., April 7th, 1894, p. 744. [24] Rif. Med., March 23rd, 1894; and Ep. B.M.J., May 5th, 1894. [25] The Polyclinic III., 17. [26] Deut. Med. Wcoh., April 19th, 1894; and Ep. B.M.J., May 5th, 1894. [27] Gazz. degli Ospitali, March 20th, 1894; and Ep. B.M.J., May 5th, 1894. [28] B.M.J., Jany. 27th, 1894, p. 188. [29] Bost. Med. and Surg. Journ., cxxix., 465. [30] B.M.J., Nov. 4th, 1893, and March 3rd, 1894. [31] B.M.J., March 3rd, 1894. [32] N. Y. Med. Jour., Feb. 5th, 1894. [33] N. Y. Med. Record, May 5th, 1894, p. 559. [34] N. Y. Med. Record, 1893, Vol. xl., No. 18 p. 552. [35] Arch. of Ped., Vol. x., No. 2, 1893; and Med. Chron., April, 1894, p. 61. [36] Med. Week., May 18th, 1894, p. 236. [37] N. Y. Med. Rec., Feb. 17th, 1894, p. 206. [38] B.M.J., April 7th, 1894, p. 745. [39] Chicago Clin. Rev., Jany., 1894. [40] B.M.J., Feb. 17th, 1894, p. 345. [41] Lancet, March 3rd, 1894, p. 537. [42] Lancet, March 10th, 1894, p. 597. [43] B.M.J., Jany. 13th, 1894, p. 63. [44] Therap. Gazette, Feb. 15th, 1894. [45] Med. Week., April 6th, 1894, p. 168. [46] Med. Gaz., March 15th, 1894, p. 173. [47] Med. Week, May 18th, 1894, p. 241. [48] Med. Rec., Jany. 27th, 1894. [49] B.M.J., April, 1894.

